# Supplementary material for: Genetic stability of Mycobacterium smegmatis under the stress of first-line antitubercular agents
Source: eLife. 2024 Nov 20;13:RP96695. doi: 10.7554/eLife.96695 (PMC11578590; doi:10.7554/eLife.96695)
Supplement: Figure 1—source data 1. [file elife-96695-fig1-data1.docx]

**Appendix 1-figure 1** Cell dimensions of *M. smegmatis* treated with different drugs

|  | Control | COMBO | INH | EMB | RIF | MMC | CIP |
| --- | --- | --- | --- | --- | --- | --- | --- |
| Mean (µm^3^) | 0.46 | 0.49 | 0.26 | 0.46 | 2.35 | 3.51 | 3.04 |
| SD | 0.25 | 0.18 | 0.13 | 0.21 | 0.88 | 1.71 | 1.14 |
| Mean length | 2.84 | 2.78 | 1.80 | 1.97 | 6.59 | 9.76 | 11.09 |
| SD | 0.92 | 0.70 | 0.54 | 0.78 | 2.41 | 4.58 | 4.04 |
| Mean width | 0.44 | 0.47 | 0.41 | 0.55 | 0.68 | 0.68 | 0.59 |
| SD | 0.08 | 0.05 | 0.07 | 0.14 | 0.09 | 0.11 | 0.10 |
| N | 212 | 100 | 84 | 91 | 95 | 118 | 137 |
