# Supplementary material for: Genetic stability of Mycobacterium smegmatis under the stress of first-line antitubercular agents
Source: eLife. 2024 Nov 20;13:RP96695. doi: 10.7554/eLife.96695 (PMC11578590; doi:10.7554/eLife.96695)
Supplement: Figure 2—source data 1. [file elife-96695-fig2-data1.docx]

| **Strain** | **Mutation rate (site/gen)** | **SD** |
| --- | --- | --- |
| Mock | 1.12E-10 | 1.53E-10 |
| UV | 2.31E-08 | 2.04E-08 |
| COMBO | <3.88E-11 | 0 |
| Isoniazid | 1.24E-10 | 1.24E-10 |
| Ethambutol | <3.88E-11 | 0 |
| Rifampicine | 7.76E-11 | 1.2E-10 |
| Mitomycin-C | 1.66E-10 | 1.9E-10 |
| Ciprofloxacin | 2.9E-10 | 2.59E-10 |

**Figure 2-source data 1:** Numerical data for mutation rates of wild-type *M. smegmatis mc^2^155* strains under antibiotic pressure.

Mutation rates determined through genome sequencing of the drug treated cells as an output of the MA process. UV(+) serves as a control reference for DNA damage. Columns represent averages, and error bars indicate the standard deviations of three individually sequenced samples.
