# Supplementary material for: Genetic stability of Mycobacterium smegmatis under the stress of first-line antitubercular agents
Source: eLife. 2024 Nov 20;13:RP96695. doi: 10.7554/eLife.96695 (PMC11578590; doi:10.7554/eLife.96695)
Supplement: Figure 3—source data 1. [file elife-96695-fig3-data1.docx]

**Figure 3-source data 1** qPCR results

| Target | Treatment | Average fold change | +/- SEM | p |
| --- | --- | --- | --- | --- |
| AdnA | CIP | 14.121 | 2.286 | 0.03887 |
|  | NT | 1 | 0.199 |  |
| AhpC | CIP | 0.752 | 0.07 | 0.59957 |
|  | NT | 1 | 0.264 |  |
| AlkA | CIP | 0.586 | 0.06 | 0.03689 |
|  | NT | 1 | 0.507 |  |
| Dcd:dut | CIP | 1.686 | 0.16 | 0.16879 |
|  | NT | 1 | 0.222 |  |
| DinB1 | CIP | 0.935 | 0.148 | 0.74175 |
|  | NT | 1 | 0.478 |  |
| DinB2 | CIP | 12.686 | 1.695 | 0.00768 |
|  | NT | 1 | 0.2 |  |
| DNA ligase | CIP | 3.171 | 0.156 | 0.05422 |
|  | NT | 1 | 0.209 |  |
| DnaE2 | CIP | 10.403 | 1.345 | 0.01572 |
|  | NT | 1 | 0.2 |  |
| Dut | CIP | 0.749 | 0.048 | 0.68244 |
|  | NT | 1 | 0.347 |  |
| End | CIP | 3.685 | 0.43 | 0.19237 |
|  | NT | 1 | 0.213 |  |
| KatG1 | CIP | 1.422 | 0.131 | 0.38096 |
|  | NT | 1 | 0.228 |  |
| LexA | CIP | 13.34 | 1.259 | 0.00578 |
|  | NT | 1 | 0.2 |  |
| Mfd | CIP | 3.833 | 0.267 | 0.03121 |
|  | NT | 1 | 0.203 |  |
| Mpg | CIP | 0.373 | 0.039 | 0.00387 |
|  | NT | 1 | 0.411 |  |
| MutM1 | CIP | 0.601 | 0.047 | 0.12714 |
|  | NT | 1 | 0.274 |  |
| MutT1 | CIP | 0.663 | 0.082 | 0.37964 |
|  | NT | 1 | 0.342 |  |
| MutT2 | CIP | 0.386 | 0.039 | 0.00986 |
|  | NT | 1 | 0.705 |  |
| MutT3 | CIP | 1.06 | 0.095 | 0.76275 |
|  | NT | 1 | 0.442 |  |
| MutT4 | CIP | 1.899 | 0.236 | 0.11622 |
|  | NT | 1 | 0.252 |  |
| MutY | CIP | 0.717 | 0.191 | 0.71034 |
|  | NT | 1 | 0.35 |  |
| Nei1 | CIP | 0.754 | 0.067 | 0.73054 |
|  | NT | 1 | 0.249 |  |
| Nei2 | CIP | 0.769 | 0.066 | 0.47785 |
|  | NT | 1 | 0.273 |  |
| NucS | CIP | 1.028 | 0.106 | 0.90935 |
|  | NT | 1 | 0.405 |  |
| Ogt | CIP | 1.307 | 0.141 | 0.60338 |
|  | NT | 1 | 0.264 |  |
| PolA | CIP | 3.768 | 0.297 | 0.02211 |
|  | NT | 1 | 0.204 |  |
| RecA | CIP | 7.496 | 0.788 | 0.00229 |
|  | NT | 1 | 0.198 |  |
| RecX | CIP | 6.234 | 0.585 | 0.00557 |
|  | NT | 1 | 0.204 |  |
| TagA | CIP | 4.436 | 0.56 | 0.0077 |
|  | NT | 1 | 0.203 |  |
| ThyA | CIP | 0.396 | 0.032 | 0.0006 |
|  | NT | 1 | 0.392 |  |
| ThyX | CIP | 0.973 | 0.061 | 0.99138 |
|  | NT | 1 | 0.231 |  |
| UdgB | CIP | 1.036 | 0.119 | 0.85443 |
|  | NT | 1 | 0.288 |  |
| UdgX | CIP | 1.177 | 0.065 | 0.76245 |
|  | NT | 1 | 0.324 |  |
| Ung | CIP | 0.374 | 0.043 | 0.0091 |
|  | NT | 1 | 0.846 |  |
| UvrA | CIP | 1.982 | 0.159 | 0.07156 |
|  | NT | 1 | 0.209 |  |
| UvrB | CIP | 5.506 | 0.288 | 0.02087 |
|  | NT | 1 | 0.202 |  |
| UvrC | CIP | 0.874 | 0.071 | 0.20758 |
|  | NT | 1 | 0.233 |  |
| UvrD | CIP | 2.452 | 0.145 | 0.02696 |
|  | NT | 1 | 0.202 |  |
| XthA | CIP | 1.101 | 0.088 | 0.83909 |
|  | NT | 1 | 0.229 |  |
| AdnA | MMC | 7.902 | 1.464 | 0.05261 |
|  | NT | 1 | 0.098 |  |
| AhpC | MMC | 0.716 | 0.086 | 0.83256 |
|  | NT | 1 | 0.115 |  |
| AlkA | MMC | 0.677 | 0.105 | 0.69495 |
|  | NT | 1 | 0.167 |  |
| Dcd:dut | MMC | 0.933 | 0.144 | 0.9583 |
|  | NT | 1 | 0.111 |  |
| DinB1 | MMC | 1.179 | 0.335 | 0.78523 |
|  | NT | 1 | 0.28 |  |
| DinB2 | MMC | 7.513 | 1.366 | 0.0094 |
|  | NT | 1 | 0.098 |  |
| DNA ligase | MMC | 1.991 | 0.357 | 0.34265 |
|  | NT | 1 | 0.102 |  |
| DnaE2 | MMC | 8.476 | 1.782 | 0.00215 |
|  | NT | 1 | 0.099 |  |
| Dut | MMC | 0.575 | 0.105 | 0.54121 |
|  | NT | 1 | 0.133 |  |
| End | MMC | 2.263 | 0.473 | 0.0107 |
|  | NT | 1 | 0.119 |  |
| KatG1 | MMC | 1.018 | 0.124 | 0.86562 |
|  | NT | 1 | 0.117 |  |
| LexA | MMC | 6.272 | 1.515 | 0.19399 |
|  | NT | 1 | 0.099 |  |
| Mfd | MMC | 1.417 | 0.229 | 0.4306 |
|  | NT | 1 | 0.104 |  |
| Mpg | MMC | 0.687 | 0.097 | 0.11023 |
|  | NT | 1 | 0.312 |  |
| MutM1 | MMC | 0.633 | 0.078 | 0.64028 |
|  | NT | 1 | 0.123 |  |
| MutT1 | MMC | 0.868 | 0.127 | 0.90405 |
|  | NT | 1 | 0.126 |  |
| MutT2 | MMC | 0.602 | 0.07 | 0.5445 |
|  | NT | 1 | 0.162 |  |
| MutT3 | MMC | 0.725 | 0.118 | 0.8164 |
|  | NT | 1 | 0.23 |  |
| MutT4 | MMC | 0.807 | 0.127 | 0.84788 |
|  | NT | 1 | 0.154 |  |
| MutY | MMC | 0.659 | 0.192 | 0.68625 |
|  | NT | 1 | 0.248 |  |
| Nei1 | MMC | 0.659 | 0.115 | 0.7256 |
|  | NT | 1 | 0.175 |  |
| Nei2 | MMC | 1.373 | 0.183 | 0.79159 |
|  | NT | 1 | 0.122 |  |
| NucS | MMC | 0.902 | 0.208 | 0.98398 |
|  | NT | 1 | 0.194 |  |
| Ogt | MMC | 0.72 | 0.1 | 0.9951 |
|  | NT | 1 | 0.182 |  |
| PolA | MMC | 1.353 | 0.233 | 0.2856 |
|  | NT | 1 | 0.108 |  |
| RecA | MMC | 6.686 | 0.934 | 0.00115 |
|  | NT | 1 | 0.098 |  |
| RecX | MMC | 5.343 | 0.81 | 0.09002 |
|  | NT | 1 | 0.099 |  |
| TagA | MMC | 1.931 | 0.517 | 0.29158 |
|  | NT | 1 | 0.108 |  |
| ThyA | MMC | 0.614 | 0.089 | 0.65887 |
|  | NT | 1 | 0.145 |  |
| ThyX | MMC | 0.56 | 0.067 | 0.66447 |
|  | NT | 1 | 0.143 |  |
| UdgB | MMC | 0.989 | 0.203 | 0.89605 |
|  | NT | 1 | 0.178 |  |
| UdgX | MMC | 0.854 | 0.192 | 0.94767 |
|  | NT | 1 | 0.122 |  |
| Ung | MMC | 0.505 | 0.116 | 0.42393 |
|  | NT | 1 | 0.369 |  |
| UvrA | MMC | 1.057 | 0.203 | 0.82833 |
|  | NT | 1 | 0.157 |  |
| UvrB | MMC | 2.065 | 0.278 | 0.21923 |
|  | NT | 1 | 0.136 |  |
| UvrC | MMC | 0.769 | 0.091 | 0.79579 |
|  | NT | 1 | 0.133 |  |
| UvrD | MMC | 1.604 | 0.218 | 0.16877 |
|  | NT | 1 | 0.102 |  |
| XthA | MMC | 0.572 | 0.156 | 0.37319 |
|  | NT | 1 | 0.21 |  |
| AdnA | COMBO | 1.151 | 0.398 | 0.99589 |
|  | NT | 1 | 1.787 |  |
| AhpC | COMBO | 1.263 | 0.253 | 0.40096 |
|  | NT | 1 | 1.069 |  |
| AlkA | COMBO | 8.576 | 1.051 | 0.03138 |
|  | NT | 1 | 0.155 |  |
| Dcd:dut | COMBO | 1.972 | 0.305 | 0.21211 |
|  | NT | 1 | 0.549 |  |
| DinB1 | COMBO | 2.095 | 0.588 | 0.69326 |
|  | NT | 1 | 1.429 |  |
| DinB2 | COMBO | 3.525 | 0.738 | 0.02779 |
|  | NT | 1 | 0.858 |  |
| DNA ligase | COMBO | 1.85 | 0.271 | 0.33174 |
|  | NT | 1 | 0.789 |  |
| DnaE2 | COMBO | 5.629 | 1.384 | 0.00555 |
|  | NT | 1 | 0.303 |  |
| Dut | COMBO | 2.309 | 0.43 | 0.21693 |
|  | NT | 1 | 0.274 |  |
| End | COMBO | 2.559 | 0.399 | 0.05372 |
|  | NT | 1 | 0.502 |  |
| KatG1 | COMBO | 17.166 | 2.578 | 0.01456 |
|  | NT | 1 | 0.115 |  |
| LexA | COMBO | 0.851 | 0.167 | 0.66476 |
|  | NT | 1 | 1.692 |  |
| Mfd | COMBO | 1.505 | 0.344 | 0.25422 |
|  | NT | 1 | 0.647 |  |
| Mpg | COMBO | 5.55 | 1.388 | 0.00063 |
|  | NT | 1 | 0.184 |  |
| MutM1 | COMBO | 4.033 | 0.577 | 0.04938 |
|  | NT | 1 | 0.205 |  |
| MutT1 | COMBO | 1.859 | 0.373 | 0.11133 |
|  | NT | 1 | 0.599 |  |
| MutT2 | COMBO | 6.075 | 0.676 | 0.02665 |
|  | NT | 1 | 0.263 |  |
| MutT3 | COMBO | 1.932 | 0.314 | 0.09768 |
|  | NT | 1 | 1.018 |  |
| MutT4 | COMBO | 1.129 | 0.304 | 0.80645 |
|  | NT | 1 | 1.525 |  |
| MutY | COMBO | 2.661 | 0.906 | 0.20534 |
|  | NT | 1 | 0.429 |  |
| Nei1 | COMBO | 2.621 | 0.541 | 0.117 |
|  | NT | 1 | 0.545 |  |
| Nei2 | COMBO | 2.029 | 0.413 | 0.3601 |
|  | NT | 1 | 0.6 |  |
| NucS | COMBO | 2.894 | 0.467 | 0.10342 |
|  | NT | 1 | 0.335 |  |
| Ogt | MMC | 4.981 | 0.553 | 0.0046 |
|  | NT | 1 | 0.182 |  |
| PolA | MMC | 1.793 | 0.285 | 0.19061 |
|  | NT | 1 | 1.276 |  |
| RecA | COMBO | 0.707 | 0.062 | 0.65932 |
|  | NT | 1 | 0.871 |  |
| RecX | COMBO | 1.323 | 0.322 | 0.50598 |
|  | NT | 1 | 0.624 |  |
| TagA | COMBO | 2.222 | 0.341 | 0.06179 |
|  | NT | 1 | 1.072 |  |
| ThyA | COMBO | 1.671 | 0.274 | 0.20173 |
|  | NT | 1 | 1.409 |  |
| ThyX | COMBO | 2.409 | 0.356 | 0.15855 |
|  | NT | 1 | 1.014 |  |
| UdgB | COMBO | 5.429 | 0.486 | 0.00956 |
|  | NT | 1 | 0.285 |  |
| UdgX | COMBO | 1.292 | 0.21 | 0.61771 |
|  | NT | 1 | 1.21 |  |
| Ung | COMBO | 0.809 | 0.269 | 0.78984 |
|  | NT | 1 | 2.765 |  |
| UvrA | COMBO | 0.95 | 0.115 | 0.70907 |
|  | NT | 1 | 1.916 |  |
| UvrB | COMBO | 1.789 | 0.28 | 0.13036 |
|  | NT | 1 | 0.78 |  |
| UvrC | COMBO | 2.551 | 0.259 | 0.1212 |
|  | NT | 1 | 0.264 |  |
| UvrD | COMBO | 1.987 | 0.251 | 0.06771 |
|  | NT | 1 | 0.38 |  |
| XthA | COMBO | 3.548 | 0.923 | 0.00032 |
|  | NT | 1 | 0.541 |  |
| AdnA | INH | 0.507 | 0.307 | 0.7943 |
|  | NT | 1 | 0.773 |  |
| AhpC | INH | 0.416 | 0.159 | 0.19812 |
|  | NT | 1 | 0.298 |  |
| AlkA | INH | 2.024 | 0.778 | 0.40531 |
|  | NT | 1 | 0.142 |  |
| Dcd:dut | INH | 0.711 | 0.363 | 0.33358 |
|  | NT | 1 | 0.198 |  |
| DinB1 | INH | 1.855 | 1.13 | 0.72874 |
|  | NT | 1 | 0.208 |  |
| DinB2 | INH | 0.833 | 0.45 | 0.5528 |
|  | NT | 1 | 0.291 |  |
| DNA ligase | INH | 0.255 | 0.118 | 0.1147 |
|  | NT | 1 | 0.607 |  |
| DnaE2 | INH | 2.006 | 0.771 | 0.87827 |
|  | NT | 1 | 0.172 |  |
| Dut | INH | 0.341 | 0.141 | 0.07244 |
|  | NT | 1 | 0.496 |  |
| End | INH | 0.618 | 0.262 | 0.3347 |
|  | NT | 1 | 0.272 |  |
| KatG1 | INH | 0.252 | 0.106 | 0.02727 |
|  | NT | 1 | 0.691 |  |
| LexA | INH | 0.846 | 0.345 | 0.87991 |
|  | NT | 1 | 0.225 |  |
| Mfd | INH | 0.434 | 0.164 | 0.17385 |
|  | NT | 1 | 0.342 |  |
| Mpg | INH | 0.344 | 0.257 | 0.85723 |
|  | NT | 1 | 0.763 |  |
| MutM1 | INH | 0.444 | 0.179 | 0.19173 |
|  | NT | 1 | 0.376 |  |
| MutT1 | INH | 0.23 | 0.092 | 0.12512 |
|  | NT | 1 | 0.659 |  |
| MutT2 | INH | 1.074 | 0.446 | 0.45787 |
|  | NT | 1 | 0.232 |  |
| MutT3 | INH | 0.565 | 0.233 | 0.27115 |
|  | NT | 1 | 0.407 |  |
| MutT4 | INH | 0.6 | 0.266 | 0.52969 |
|  | NT | 1 | 0.278 |  |
| MutY | INH | 0.249 | 0.108 | 0.13517 |
|  | NT | 1 | 0.668 |  |
| Nei1 | INH | 0.428 | 0.176 | 0.10021 |
|  | NT | 1 | 0.541 |  |
| Nei2 | INH | 0.178 | 0.087 | 0.00101 |
|  | NT | 1 | 0.731 |  |
| NucS | INH | 1.184 | 0.574 | 0.8894 |
|  | NT | 1 | 0.228 |  |
| Ogt | INH | 1.436 | 0.672 | 0.51484 |
|  | NT | 1 | 0.142 |  |
| PolA | INH | 0.403 | 0.164 | 0.18206 |
|  | NT | 1 | 0.339 |  |
| RecA | INH | 0.726 | 0.278 | 0.53931 |
|  | NT | 1 | 0.197 |  |
| RecX | INH | 0.797 | 0.305 | 0.86511 |
|  | NT | 1 | 0.195 |  |
| TagA | INH | 0.221 | 0.094 | 0.08878 |
|  | NT | 1 | 0.55 |  |
| ThyA | INH | 0.04 | 0.021 | 0.01628 |
|  | NT | 1 | 6.6 |  |
| ThyX | INH | 0.208 | 0.106 | 0.18768 |
|  | NT | 1 | 0.803 |  |
| UdgB | INH | 0.622 | 0.24 | 0.19842 |
|  | NT | 1 | 0.146 |  |
| UdgX | INH | 0.371 | 0.143 | 0.35299 |
|  | NT | 1 | 0.262 |  |
| Ung | INH | 0.516 | 0.196 | 0.33083 |
|  | NT | 1 | 0.236 |  |
| UvrA | INH | 0.729 | 0.301 | 0.89194 |
|  | NT | 1 | 0.212 |  |
| UvrB | INH | 0.749 | 0.299 | 0.99766 |
|  | NT | 1 | 0.258 |  |
| UvrC | INH | 0.185 | 0.08 | 0.01813 |
|  | NT | 1 | 0.753 |  |
| UvrD | INH | 0.362 | 0.136 | 0.02415 |
|  | NT | 1 | 0.505 |  |
| XthA | INH | 0.741 | 0.339 | 0.39897 |
|  | NT | 1 | 0.282 |  |
| AdnA | EMB | 1.203 | 0.141 | 0.86275 |
|  | NT | 1 | 0.185 |  |
| AhpC | EMB | 0.568 | 0.062 | 0.6683 |
|  | NT | 1 | 0.206 |  |
| AlkA | EMB | 0.844 | 0.087 | 0.72902 |
|  | NT | 1 | 0.265 |  |
| Dcd:dut | EMB | 1.269 | 0.128 | 0.87932 |
|  | NT | 1 | 0.228 |  |
| DinB1 | EMB | 0.324 | 0.073 | 0.10778 |
|  | NT | 1 | 2.273 |  |
| DinB2 | EMB | 1.515 | 0.173 | 0.40975 |
|  | NT | 1 | 0.367 |  |
| DNA ligase | EMB | 1.705 | 0.256 | 0.74016 |
|  | NT | 1 | 0.159 |  |
| DnaE2 | EMB | 0.818 | 0.13 | 0.73481 |
|  | NT | 1 | 0.497 |  |
| Dut | EMB | 0.295 | 0.035 | 0.18335 |
|  | NT | 1 | 0.547 |  |
| End | EMB | 1.214 | 0.127 | 0.30429 |
|  | NT | 1 | 0.489 |  |
| KatG1 | EMB | 0.237 | 0.038 | 0.23333 |
|  | NT | 1 | 0.71 |  |
| LexA | EMB | 2.047 | 0.193 | 0.80759 |
|  | NT | 1 | 0.193 |  |
| Mfd | EMB | 1.12 | 0.141 | 0.9215 |
|  | NT | 1 | 0.234 |  |
| Mpg | EMB | 1.054 | 0.279 | 0.73747 |
|  | NT | 1 | 0.631 |  |
| MutM1 | EMB | 0.268 | 0.043 | 0.06411 |
|  | NT | 1 | 0.42 |  |
| MutT1 | EMB | 0.463 | 0.059 | 0.54076 |
|  | NT | 1 | 0.567 |  |
| MutT2 | EMB | 0.311 | 0.057 | 0.2705 |
|  | NT | 1 | 1.246 |  |
| MutT3 | EMB | 0.243 | 0.034 | 0.26496 |
|  | NT | 1 | 0.758 |  |
| MutT4 | EMB | 1.004 | 0.147 | 0.96784 |
|  | NT | 1 | 0.398 |  |
| MutY | EMB | 0.84 | 0.081 | 0.77104 |
|  | NT | 1 | 0.356 |  |
| Nei1 | EMB | 0.175 | 0.043 | 0.18413 |
|  | NT | 1 | 2.692 |  |
| Nei2 | EMB | 0.305 | 0.035 | 0.28952 |
|  | NT | 1 | 1.048 |  |
| NucS | EMB | 1.133 | 0.113 | 0.7434 |
|  | NT | 1 | 0.263 |  |
| Ogt | EMB | 1.84 | 0.19 | 0.42958 |
|  | NT | 1 | 0.19 |  |
| PolA | EMB | 1.064 | 0.116 | 0.85379 |
|  | NT | 1 | 0.227 |  |
| RecA | EMB | 1.413 | 0.154 | 0.08018 |
|  | NT | 1 | 0.286 |  |
| RecX | EMB | 1.665 | 0.281 | 0.73135 |
|  | NT | 1 | 0.142 |  |
| TagA | EMB | 0.902 | 0.101 | 0.83606 |
|  | NT | 1 | 0.294 |  |
| ThyA | EMB | 0.202 | 0.024 | 0.31757 |
|  | NT | 1 | 0.688 |  |
| ThyX | EMB | 0.526 | 0.071 | 0.40416 |
|  | NT | 1 | 0.342 |  |
| UdgB | EMB | 3.51 | 0.378 | 0.33896 |
|  | NT | 1 | 0.18 |  |
| UdgX | EMB | 0.431 | 0.057 | 0.08724 |
|  | NT | 1 | 0.599 |  |
| Ung | EMB | 2.938 | 0.618 | 0.79681 |
|  | NT | 1 | 0.109 |  |
| UvrA | EMB | 0.878 | 0.114 | 0.82035 |
|  | NT | 1 | 0.378 |  |
| UvrB | EMB | 1.129 | 0.134 | 0.98668 |
|  | NT | 1 | 0.316 |  |
| UvrC | EMB | 0.47 | 0.061 | 0.29949 |
|  | NT | 1 | 0.434 |  |
| UvrD | EMB | 0.174 | 0.025 | 0.13545 |
|  | NT | 1 | 0.627 |  |
| XthA | EMB | 1.478 | 0.287 | 0.76146 |
|  | NT | 1 | 0.245 |  |
| AdnA | RIF | 2.277 | 0.204 | 0.1663 |
|  | NT | 1 | 0.122 |  |
| AhpC | RIF | 0.228 | 0.029 | 0.04564 |
|  | NT | 1 | 0.272 |  |
| AlkA | RIF | 2.122 | 0.14 | 0.63226 |
|  | NT | 1 | 0.115 |  |
| Dcd:dut | RIF | 0.665 | 0.083 | 0.66736 |
|  | NT | 1 | 0.266 |  |
| DinB1 | RIF | 0.695 | 0.177 | 0.48363 |
|  | NT | 1 | 0.273 |  |
| DinB2 | RIF | 1.379 | 0.138 | 0.15913 |
|  | NT | 1 | 0.129 |  |
| DNA ligase | RIF | 0.958 | 0.139 | 0.12799 |
|  | NT | 1 | 0.109 |  |
| DnaE2 | RIF | 0.66 | 0.028 | 1 |
|  | NT | 1 | 0.184 |  |
| Dut | RIF | 0.878 | 0.046 | 0.87121 |
|  | NT | 1 | 0.109 |  |
| End | RIF | 0.487 | 0.105 | 0.54775 |
|  | NT | 1 | 0.527 |  |
| KatG1 | RIF | 0.806 | 0.063 | 0.95255 |
|  | NT | 1 | 0.113 |  |
| LexA | RIF | 0.848 | 0.37 | 0.71664 |
|  | NT | 1 | 0.357 |  |
| Mfd | RIF | 1.38 | 0.075 | 0.6808 |
|  | NT | 1 | 0.105 |  |
| Mpg | RIF | 1.369 | 0.08 | 0.03145 |
|  | NT | 1 | 0.192 |  |
| MutM1 | RIF | 0.752 | 0.027 | 0.74627 |
|  | NT | 1 | 0.124 |  |
| MutT1 | RIF | 0.895 | 0.088 | 0.79417 |
|  | NT | 1 | 0.113 |  |
| MutT2 | RIF | 0.395 | 0.035 | 0.11781 |
|  | NT | 1 | 0.343 |  |
| MutT3 | RIF | 0.392 | 0.064 | 0.26692 |
|  | NT | 1 | 0.374 |  |
| MutT4 | RIF | 2.146 | 0.337 | 0.91444 |
|  | NT | 1 | 0.181 |  |
| MutY | RIF | 0.894 | 0.132 | 0.66026 |
|  | NT | 1 | 0.226 |  |
| Nei1 | RIF | 0.744 | 0.079 | 0.68867 |
|  | NT | 1 | 0.443 |  |
| Nei2 | RIF | 0.751 | 0.119 | 0.65362 |
|  | NT | 1 | 0.303 |  |
| NucS | RIF | 0.929 | 0.046 | 0.81321 |
|  | NT | 1 | 0.147 |  |
| Ogt | RIF | 3.192 | 0.422 | 0.18641 |
|  | NT | 1 | 0.098 |  |
| PolA | RIF | 1.683 | 0.298 | 0.67136 |
|  | NT | 1 | 0.118 |  |
| RecA | RIF | 0.754 | 0.043 | 0.48499 |
|  | NT | 1 | 0.162 |  |
| RecX | RIF | 0.598 | 0.055 | 0.11886 |
|  | NT | 1 | 0.158 |  |
| TagA | RIF | 0.804 | 0.094 | 0.79958 |
|  | NT | 1 | 0.208 |  |
| ThyA | RIF | 0.462 | 0.041 | 0.5 |
|  | NT | 1 | 0.172 |  |
| ThyX | RIF | 0.421 | 0.061 | 0.5 |
|  | NT | 1 | 0.362 |  |
| UdgB | RIF | 1.687 | 0.195 | 0.25647 |
|  | NT | 1 | 0.11 |  |
| UdgX | RIF | 3.289 | 0.129 | 0.55937 |
|  | NT | 1 | 0.088 |  |
| Ung | RIF | 0.993 | 0.174 | 0.97323 |
|  | NT | 1 | 0.108 |  |
| UvrA | RIF | 1.39 | 0.056 | 0.83498 |
|  | NT | 1 | 0.133 |  |
| UvrB | RIF | 1.453 | 0.098 | 0.8279 |
|  | NT | 1 | 0.103 |  |
| UvrC | RIF | 0.423 | 0.027 | 0.12612 |
|  | NT | 1 | 0.266 |  |
| UvrD | RIF | 0.771 | 0.029 | 0.69513 |
|  | NT | 1 | 0.093 |  |
| XthA | RIF | 1.035 | 0.053 | 0.90709 |
|  | NT | 1 | 0.103 |  |
