# Supplementary material for: Genetic stability of Mycobacterium smegmatis under the stress of first-line antitubercular agents
Source: eLife. 2024 Nov 20;13:RP96695. doi: 10.7554/eLife.96695 (PMC11578590; doi:10.7554/eLife.96695)
Supplement: Figure 4—source data 1. [file elife-96695-fig4-data1.docx]

**Figure 4-source data 1** dNTP concentrations in cellular extracts upon treatment with drugs

| Treatment | dNTP | Sample | μM | +/-SEM | Fold change | p-value  (t-probe) |
| --- | --- | --- | --- | --- | --- | --- |
| COMBO | dGTP | Treated | 524 | 46 | 0.54 | 0.125 |
|  |  | Non-treated | 977 | 237 |  |  |
|  | dCTP | Treated | 656 | 69 | 0.98 | 0.925 |
|  |  | Non-treated | 671 | 155 |  |  |
|  | dTTP | Treated | 756 | 73 | 1.10 | 0.443 |
|  |  | Non-treated | 658 | 123 |  |  |
|  | dATP | Treated | 231 | 71 | 0.39 | 0.161 |
|  |  | Non-treated | 587 | 175 |  |  |
| INH | dGTP | Treated | 599 | 235 | 0.40 | 0.020 |
|  |  | Non-treated | 1508 | 55 |  |  |
|  | dCTP | Treated | 311 | 25 | 0.68 | 0.246 |
|  |  | Non-treated | 460 | 108 |  |  |
|  | dTTP | Treated | 458 | 130 | 0.61 | 0.194 |
|  |  | Non-treated | 748 | 156 |  |  |
|  | dATP | Treated | 403 | 99 | 0.54 | 0.038 |
|  |  | Non-treated | 748 | 87 |  |  |
| EMB | dGTP | Treated | 154 | 26 | 0.05 | 0.057 |
|  |  | Non-treated | 3025 | 675 |  |  |
|  | dCTP | Treated | 107 | 7 | 0.16 | 0.068 |
|  |  | Non-treated | 686 | 234 |  |  |
|  | dTTP | Treated | 348 | 28 | 0.20 | 0.052 |
|  |  | Non-treated | 1711 | 509 |  |  |
|  | dATP | Treated | 163 | 32 | 0.15 | 0.006 |
|  |  | Non-treated | 1062 | 98 |  |  |
| RIF | dGTP | Treated | 253 | 39 | 0.65 | 0.056 |
|  |  | Non-treated | 387 | 17 |  |  |
|  | dCTP | Treated | 202 | 33 | 3.30 | 0.013 |
|  |  | Non-treated | 60 | 6 |  |  |
|  | dTTP | Treated | 238 | 42 | 1.50 | 0.182 |
|  |  | Non-treated | 158 | 22 |  |  |
|  | dATP | Treated | 116 | 13 | 0.96 | 0.866 |
|  |  | Non-treated | 121 | 25 |  |  |
| MMC | dGTP | Treated | 476 | 37 | 0.43 | 0.035 |
|  |  | Non-treated | 1103 | 198 |  |  |
|  | dCTP | Treated | 230 | 16 | 2.20 | 0.025 |
|  |  | Non-treated | 107 | 27 |  |  |
|  | dTTP | Treated | 563 | 91 | 1.30 | 0.342 |
|  |  | Non-treated | 432 | 91 |  |  |
|  | dATP | Treated | 212 | 22 | 0.60 | 0.154 |
|  |  | Non-treated | 355 | 67 |  |  |
| CIP | dGTP | Treated | 587 | 21 | 0.43 | 0.002 |
|  |  | Non-treated | 1380 | 114 |  |  |
|  | dCTP | Treated | 371 | 20 | 1.10 | 0.311 |
|  |  | Non-treated | 336 | 47 |  |  |
|  | dTTP | Treated | 5296 | 557 | 7.00 | 0.003 |
|  |  | Non-treated | 757 | 87 |  |  |
|  | dATP | Treated | 4338 | 574 | 7.10 | 0.008 |
|  |  | Non-treated | 612 | 69 |  |  |
