# Supplementary material for: Genetic stability of Mycobacterium smegmatis under the stress of first-line antitubercular agents
Source: eLife. 2024 Nov 20;13:RP96695. doi: 10.7554/eLife.96695 (PMC11578590; doi:10.7554/eLife.96695)
Supplement: Supplementary file 1. [file elife-96695-supp1.docx]

**Supplementary Table 1** The nucleotide sequence and measured efficiency of primers used for the qPCR

| Target | Msm Gene ID | Sequence | Efficiency |
| --- | --- | --- | --- |
| AdnA_for | MSMEG_1941 | CGCAGTCCTACCGTTGC | 81.6 |
| AdnA_rev |  | CTTCGGCGTGTGTGGAG |  |
| AhpC_for | MSMEG_4753 | CCGACAAGCCCGAGAAG | 103 |
| AlhpC_rev |  | GGAACGTGGACCGGATG |  |
| AlkA_for | MSMEG_4925 | GCCTCCATCCGTCAGTTC | 83.5 |
| AlkaA_rev |  | GCCGAACAATCCCTCGTAG |  |
| Dcd:dut_for | MSMEG_0678 | CGGTTGGAGGGCAAGTC | 98.2 |
| Dcd:dut_rev |  | CACAGCTGCCCGATCTTC |  |
| DinB1_for | MSMEG_3172 | TCACGGTCAAGCTCAAGAAG | 83.2 |
| DinB1_rev |  | AACCCAACGCCCACAAG |  |
| DinB2_for | MSMEG_6443 | CCAGTTACGAGGCCAAGG | 81.2 |
| DinB2_rev |  | GGGTGGTGTCGTGGAAG |  |
| DNA ligase_for | MSMEG_2362 | GGAGGTCAAACGCAAGGG | 91.1 |
| DNA ligase_rev |  | CAACTCGGTTCCGCACTC |  |
| DnaE2_for | MSMEG_1633 | GCCTCGCTGGTGTTCTAC | 87.7 |
| DnaE2_rev |  | AGGCTGGCATTCACGTC |  |
| Dut_for | MSMEG_2765 | ATACCGCACGGAATGGTC | 97.1 |
| Dut_rev |  | GTCTGCGGATCCAGGTTG |  |
| End_for | MSMEG_1383 | AGCGCATCAAGTCCATCAC | 83 |
| End_rev |  | GCCGTCTCGCAGATCAC |  |
| Ffh_for | MSMEG_2430 | GAGCTCATCGGCATCCTC | 86.2 |
| Ffh_rev |  | GGGCTGTGACCCTTGTC |  |
| KatG1_for | MSMEG_6384 | GCCCATCGGAGAAGCTC | 96 |
| KatG1_rev |  | CCTCGTCCAGCGGATTG |  |
| LexA_for | MSMEG_2740 | GTTCCTGCTCAAGGTCGTC | 105 |
| LexA_rev |  | CATGAGCCACACCTGACC |  |
| Mfd_for | MSMEG_5423 | GAGCTCACCCGGTTCAC | 89 |
| Mfd_rev |  | CTTGGTGGCGGTCTTCTC |  |
| Mpg_for | MSMEG_3759 | GTGCGGAACTCGGTGATG | 89.6 |
| Mpg_rev |  | GCTGTGCACTGTCGACTC |  |
| MutM1_for | MSMEG_2419 | CAGGCGCGGAAAGTACC | 93 |
| MutM1_rev |  | CGCTGATCGACGAAGCTC |  |
| MutT1_for | MSMEG_2390 | GGTGGACAAGCTCGTATGG | 107 |
| MutT1_rev |  | GCGATCGTCACCCTTGTAG |  |
| MutT2_for | MSMEG_5148 | GGCTGTGGGAACTTCCTG | 86.7 |
| MutT2_rev |  | TGTCATGGCGTCGTTGAG |  |
| MutT3_for | MSMEG_0790 | CGTACACGACGGTGATCG | 92.1 |
| MutT3_rev |  | GTAGGCGCTGCCAACTC |  |
| MutT4_for | MSMEG_6927 | CAGGCGGTCTGGTCATC | 87.1 |
| MutT4_rev |  | TGGATCCCGGTCTCCTC |  |
| MutY_for | MSMEG_6083 | GGGAAAGCTCGGCTACC | 86.4 |
| MutY_rev |  | GGAACGCTCGCCTGATAG |  |
| Nei1_for | MSMEG_4683 | CATCGGCGCTCAGTACG | 99.7 |
| Nei1_rev |  | CGGTCTGCGTGACTTGG |  |
| Nei2_for | MSMEG_1756 | GCGGTACCGACATGGAC | 92.1 |
| Nei2_rev |  | GAAACACAACTCGTTGCAGTAG |  |
| NucS_for | MSMEG_4923 | CGCGCTACCTGGAACTG | 99.7 |
| NucS_rev |  | GTACTCGTCGCTGTCCATTC |  |
| Ogt_for | MSMEG_4928 | AGATCCCGTACGGACAGAC | 92.6 |
| Ogt_rev |  | CCCATAACCCGTGAGACTTC |  |
| PolA_for | MSMEG_3839 | GAGCTCACCCGGTTCAC | 83.9 |
| PolA_rev |  | CTTGGTGGCGGTCTTCTC |  |
| ProC_for | MSMEG_0943 | GCCCGGCGTACTTCTTC | 73.3 |
| ProC_rev |  | CGGCGTTCACCTGATCC |  |
| RecA_for | MSMEG_2723 | CGCGTCAAGGTCGTCAAG | 90.1 |
| RecA_rev |  | ACCCTCGTAGGTGAACCAG |  |
| RecX_for | MSMEG_2724 | CTCGAAACCCAGCTGACC | 89.9 |
| RecX_rev |  | GAGCTCGACAGCCAAGG |  |
| SigA_for | MSMEG_2758 | CATCTGCTGGAGGCGAAC | 87.7 |
| SigA_rev |  | CTTGTAGCCCTTGGTGTAGTC |  |
| TagA_for | MSMEG_5082 | GACTACCACGACACCGAATG | 91.4 |
| TagA_rev |  | GGATCGAACCCGTGGAAC |  |
| ThyA_for | MSMEG_2670 | GTCGGGTGAGCACATCG | 92.8 |
| ThyA_rev |  | GGCGACGTAGAACTGGAAG |  |
| ThyX_for | MSMEG_2683 | CGTACAGCTGATCGCCAAG | 94.2 |
| ThyX_rev |  | CGTTGGTCGCGGTCTTC |  |
| UdgB_for | MSMEG_5031 | ACGTTGACCACCGCATAC | 97.7 |
| UdgB_rev |  | TTCGTTCCCGATCTGCTTG |  |
| UdgX_for | MSMEG_0265 | CCCGGTGACAAAGAGGAC | 91.8 |
| UdgX_rev |  | CTTGTGGATGCGTCGTTTG |  |
| Ung_for | MSMEG_2399 | CCGTGGCAGATCAGGTG | 89.2 |
| Ung_rev |  | TGTCGGGTAGGGATCCTG |  |
| UvrA_for | MSMEG_3808 | CCTGGGCATCCGCAAAG | 94.6 |
| UvrA_rev |  | CCCATCTCCTCACCGAGAC |  |
| UvrB_for | MSMEG_3816 | GAGAAGGACAGCTCGATCAAC | 96.6 |
| UvrB_rev |  | CTGACCCACCTGCAACTC |  |
| UvrC_for | MSMEG_3078 | TCGATTTCTGCGACTTCCTG | 88.1 |
| UvrC_rev |  | CCACGGCCTGTTTCTCC |  |
| UvrD_for | MSMEG_5534 | GGGAGGACGGCATGTTC | 86.3 |
| UvrD_rev |  | CGCGATTCCGGGTTGAG |  |
| XthA_for | MSMEG_0829 | CCTACCACGGGCTCAAC | 80.7 |
| XthA_rev |  | GCACGTAGAGGCTCCATAC |  |
