# Supplementary material for: Genetic stability of Mycobacterium smegmatis under the stress of first-line antitubercular agents
Source: eLife. 2024 Nov 20;13:RP96695. doi: 10.7554/eLife.96695 (PMC11578590; doi:10.7554/eLife.96695)
Supplement: Supplementary file 2. [file elife-96695-supp2.docx]

**Supplementary Table 2** User Guide for Whole Genome Sequencing (WGS) Data Files Deposited in the European Nucleotide Archive (ENA)

| Experiment | Treatment | Sample | Sample description | ENA file name | Experiment length |
| --- | --- | --- | --- | --- | --- |
| MA | Ciprofloxacin | CIP1 | 5 different lineages of ciprofloxacin treated Mycobacterium smegmatis | CipA.rg.bam | 60 days |
| MA |  | CIP2 | 5 different lineages of ciprofloxacin treated Mycobacterium smegmatis | CipB.rg.bam | 60 days |
| MA |  | CIP3 | 5 different lineages of ciprofloxacin treated Mycobacterium smegmatis | CipC.rg.bam | 60 days |
| MA | Rifampicin | RIF1 | 6 different lineages of rifampicin treated Mycobacterium smegmatis | RIFA.rg.bam | 60 days |
| MA |  | RIF2 | 5 different lineages of rifampicin treated Mycobacterium smegmatis | RIFB.rg.bam | 60 days |
| MA |  | RIF3 | 5 different lineages of rifampicin treated Mycobacterium smegmatis | RIFC.rg.bam | 60 days |
| MA | Isoniazid | INH1 | 5 different lineages of isoniazid treated Mycobacterium smegmatis | INH1.rg.bam | 60 days |
| MA |  | INH2 | 5 different lineages of isoniazid treated Mycobacterium smegmatis | INH2.rg.bam | 60 days |
| MA |  | INH3 | 5 different lineages of isoniazid treated Mycobacterium smegmatis | INHA.rg.bam | 60 days |
| MA | Ethambutol | EMB1 | 5 different lineages of ethambutol treated Mycobacterium smegmatis | EMBA.rg.bam | 60 days |
| MA |  | EMB2 | 6 different lineages of ethambutol treated Mycobacterium smegmatis | EMBB.rg.bam | 60 days |
| MA |  | EMB3 | 5 different lineages of ethambutol treated Mycobacterium smegmatis | EMBC.rg.bam | 60 days |
| MA | Combination of first line drugs | COMBO1 | 5 different lineages of pyrazinamide, ethambutol, isoniazid and rifampicin treated Mycobacterium smegmatis | CombA.rg.bam | 60 days |
| MA |  | COMBO2 | 6 different lineages of pyrazinamide, ethambutol, isoniazid and rifampicin treated Mycobacterium smegmatis | CombB.rg.bam | 60 days |
| MA |  | COMBO3 | 5 different lineages of pyrazinamide, ethambutol, isoniazid and rifampicin treated Mycobacterium smegmatis | CombC.rg.bam | 60 days |
| MA | MitomycinC | MMC1 | 5 different lineages of mitomycinC treated Mycobacterium smegmatis | MMCA.rg.bam | 60 days |
| MA |  | MMC2 | 5 different lineages of mitomycinC treated Mycobacterium smegmatis | MMCB.rg.bam | 60 days |
| MA |  | MMC3 | 5 different lineages of mitomycinC treated Mycobacterium smegmatis | MMCC.rg.bam | 60 days |
| MA | UV | UV1 | 5 different lineages of UV radiation treated Mycobacterium smegmatis | UV1.rg.bam | 60 days |
| MA |  | UV2 | 5 different lineages of UV radiation treated Mycobacterium smegmatis | UV2.rg.bam | 60 days |
| MA |  | UV3 | 6 different lineages of UV radiation treated Mycobacterium smegmatis | UV3.rg.bam | 60 days |
| MA | Control, no treatment | MOCK1 | 5 different lineages of untreated Mycobacterium smegmatis | MockA.rg.bam | 120 days |
| MA |  | MOCK2 | 5 different lineages of untreated Mycobacterium smegmatis | MockB.rg.bam | 120 days |
| MA |  | MOCK3 | 5 different lineages of untreated Mycobacterium smegmatis | MockC.rg.bam | 120 days |
| MA |  | MOCK4 | 5 different lineages of untreated Mycobacterium smegmatis | MockD.rg.bam | 120 days |
| MA |  | MOCK5 | 5 different lineages of untreated Mycobacterium smegmatis | MockE.rg.bam | 120 days |
| MA |  | MOCK6 | 5 different lineages of untreated Mycobacterium smegmatis | MockF.rg.bam | 120 days |
| MA |  | MOCK7 | 5 different lineages of untreated Mycobacterium smegmatis | MockG.rg.bam | 120 days |
| MA |  | MOCK8 | 5 different lineages of untreated Mycobacterium smegmatis | MockH.rg.bam | 120 days |
| MA |  | MOCK9 | 5 different lineages of untreated Mycobacterium smegmatis | MockI.rg.bam | 60 days |
| MA |  | MOCK10 | 6 different lineages of untreated Mycobacterium smegmatis | MockJ.rg.bam | 60 days |
| MA |  | MOCK11 | 5 different lineages of untreated Mycobacterium smegmatis | MockK.rg.bam | 60 days |
| MA |  | MOCK12 | 5 different lineages of untreated Mycobacterium smegmatis | MockL.rg.bam | 60 days |
| MA | No treatment | APAW | 1 strain, common ancestor of every treated and untreated lineages, wild-type Mycobacterium smegmatis | APAW.rg.bam | - |
| MA | No treatment | WT_MSM | 1 strain, wild-type Mycobacterium smegmatis | WT_Msm.rg.bam | - |
| Fluctuation assay | Ciprofloxacin | A03 | 5 different lineages of 0.3 µg/ml ciprofloxacin treated Mycobacterium smegmatis | A03.rg.bam | 4 days |
| Fluctuation assay | Ciprofloxacin | A05sel | 5 different lineages of 0.5 µg/ml ciprofloxacin treated Mycobacterium smegmatis | A05sel.rg.bam | 4 days |
| Fluctuation assay | Untreated | ANT | 5 different lineages of untreated Mycobacterium smegmatis | ANT.rg.bam | 4 days |
| Fluctuation assay | Untreated | At0 | 1 strain, common ancestor line of fluctuation assay treatment for samples A03, A05sel and ANT samples^1^ | At0.rg.bam | 4 days |
| Fluctuation assay | Ciprofloxacin | B03 | 5 different lineages of 0.3 µg/ml ciprofloxacin treated Mycobacterium smegmatis | B03.rg.bam | 4 days |
| Fluctuation assay | Ciprofloxacin | B05sel | 5 different lineages of 0.5 µg/ml ciprofloxacin treated Mycobacterium smegmatis | B05sel.rg.bam | 4 days |
| Fluctuation assay | Untreated | BNT | 5 different lineages of untreated Mycobacterium smegmatis | BNT.rg.bam | 4 days |
| Fluctuation assay | Untreated | Bt0 | 1 strain, common ancestor line of fluctuation assay treatment for samples B03, B05sel and BNT samples^1^ | Bt0.rg.bam | 4 days |
| Fluctuation assay | Ciprofloxacin | C03 | 5 different lineages of 0.3 µg/ml ciprofloxacin treated Mycobacterium smegmatis | C03.rg.bam | 4 days |
| Fluctuation assay | Ciprofloxacin | C05sel | 5 different lineages of 0.5 µg/ml ciprofloxacin treated Mycobacterium smegmatis | C05sel.rg.bam | 4 days |
| Fluctuation assay | Untreated | CNT | 5 different lineages of untreated Mycobacterium smegmatis | CNT.rg.bam | 4 days |
| Fluctuation assay | Untreated | Ct0 | 1 strain, common ancestor line of fluctuation assay treatment for samples C03, C05sel and CNT samples^1^ | Ct0.rg.bam | 4 days |

^1Note that At0, Bt0 and Ct0 strains also originated from APAW strain used for MA experiment.^
